# The ERP Effects of Combined Cognitive Training on Intention-Based and Stimulus-Based Actions in Older Chinese Adults

**DOI:** 10.3389/fpsyg.2016.01670

**Published:** 2016-10-27

**Authors:** Ya-Nan Niu, Xinyi Zhu, Juan Li, Jiang-Ning Fu

**Affiliations:** Center on Aging Psychology, CAS Key Laboratory of Mental Health, Institute of Psychology, Chinese Academy of SciencesBeijing, China

**Keywords:** combined cognitive training, intention-based action, stimulus-based action, executive function, associative memory, ERPs, motor cognition, older adults

## Abstract

Age-related decreases in action are caused by neuromuscular weakness and cognitive decline. Although physical interventions have been reported to have beneficial effects on cognitive function in older adults, whether cognitive training improves action-related function remains unclear. In this study, we investigated the effects of combined cognitive training on intention-based and stimulus-based actions in older adults using event-related potentials (ERPs). A total of 26 healthy older adults (16 in the training group and 10 in the control group) participated in the study. The training group received 16 sessions of cognitive training, including eight sessions of executive function training and eight sessions of memory strategy training. Before and after training, both groups of participants underwent cognitive assessments and ERP recordings during both the acquisition and test phases with a motor cognitive paradigm. During the acquisition phase, subjects were asked to press one of two keys, either using a self-selected (intention-based) method or based on the preceding stimulus (stimulus-based). During the test phase, subjects were asked to respond to the pre-cues with either congruent or incongruent tasks. Using ERP indices—including readiness potential, P3 and contingent negative variation to identify motor preparation, stimulus processing and interference effect, respectively—we revealed the effects of training on both intention-based and stimulus-based actions. The correlations were also computed between the improved cognitive performance and the ERP amplitudes. It was shown that the improved executive function might extend substantial benefits to both actions, whereas associative memory may be specifically related to the bidirectional action-effect association of intention-based action, although the training effect of memory was absent during the insufficient training hours. In sum, the present study provided empirical evidence demonstrating that action could benefit from cognitive training. Clinical Trial Registration: www.chictr.org.cn, identifier: ChiCTR-OON-16007793.

## Introduction

Age-related decreases in action are caused by neuromuscular weakness and cognitive decline (Sterr and Dean, 2008; Seidler et al., 2010 for a review). Although the function of action and cognition declines with age, it has been well documented that brain plasticity can remain in older adults (Kelly et al., 2014 for a review; Lampit et al., 2015), which provided encouraging evidence for effective interventions aiming to improve action and cognitive functions. The relationship between action and cognition has been continuously examined—for example, physical intervention has shown beneficial effects on cognitive function for older adults (Hillman et al., 2002; Colcombe and Kramer, 2003 for a review). However, the question of whether cognitive training improves action-related function remains unclear. In this study, we aimed to explore the electrophysiological effect of combined cognitive training on motor cognition in older Chinese adults.

It has been shown that the age-related decline of executive function is associated with motor deficits among older adults. On one hand, executive function has been reported to be responsible for age-related motor slowing in both the stimulus processing and motor planning stages (Sterr and Dean, 2008; Berchicci et al., 2012; Woods et al., 2015). On the other hand, exercise has been shown to benefit executive function. A meta-analytic study showed that the benefits of physical training in older adults are greatest for executive function (Colcombe and Kramer, 2003 for a review), suggesting that executive function may underpin motor-related cognitive processing in a fundamental and wide-ranging manner.

Recently, with the development and further exploration of ideomotor theory (Greenwald, 1970), it has been recognized that memory also plays an important role in motor cognition. For example, when people intended to achieve a desired goal or action effect, it was necessary to retrieve the related action through associative memory (Elsner et al., 2002). A stream of studies has revealed the related cognitive processing by comparing intention-based action (i.e., ideomotor action) with stimulus-based action (i.e., sensorimotor action) (Elsner and Hommel, 2001; Haggard et al., 2002; Waszak et al., 2005; Keller et al., 2006; Herwig et al., 2007; Herwig, 2015 for a review), which yields a reliable paradigm for the experimental investigation of intention-based action. Although intention-based action is a more complex and evolved action that plays an important role in everyday life (Herwig, 2015 for a review), stimulus-based action, which follows the simple stimulus-response rule, involves tasks that have been commonly used in past studies—such as the reaction task.

In previous behavioral studies comparing intention-based and stimulus-based action modes (Elsner and Hommel, 2001; Haggard et al., 2002; Herwig et al., 2007), participants were asked to perform tasks using an acquisition-test paradigm, which is similar to the study-test paradigm that is typically used in memory studies. In Herwig et al. (2007), during the acquisition phase, the participants in the intention-based action group were asked to make a self-selected keypress (the action) that was always followed by a certain tone with either a high or low pitch (the effect of the action), thus creating an *action-effect binding*. For the stimulus-based action group, participants were asked to make certain responses by pressing a key based on a specific rule. During the subsequent test phase, the participants were asked to make speeded keypresses in response to tones with high or low pitches based on either the congruent or incongruent rule that was learned during the acquisition phase. The results showed an interference effect of reaction time only in the intention-based action group—that is, faster performance occurred in congruent tasks than in incongruent tasks. This result was interpreted as showing that the *action-effect binding* that was acquired in the acquisition phase was bidirectional and then activated in reverse (i.e., *effect-action retrieval*) during the test phase, which postponed the reaction time when the incongruent rule was applied. However, for stimulus-based action, the interference effect of the reaction time was missing because it was based on simpler stimulus-response linkages that are monodirectional (Herwig et al., 2007). Notably, these processes of *action-effect binding* and *effect-action retrieval* resembled the processes of associative memory coding and retrieval, respectively. The involvement of associative memory in intention-based action was further improved by neuroimaging studies that revealed activations in both the supplementary motor area (SMA) and the medial temporal memory system, including hippocampus and parahippocampal gyrus, during either action image or execution (Elsner et al., 2002; Melcher et al., 2008, 2013; Pfister et al., 2014).

The different processing modes between the two types of actions have also been revealed by event-related potentials (ERPs) (Waszak et al., 2005; Keller et al., 2006). The results showed that during the acquisition phase, the readiness potential (RP) (e.g., Shibasaki and Hallett, 2006) component, which is a slow, negative going potential prior to an action execution that reflects the general preparation of voluntary movement, was more negative under intention-based conditions, but the P3 component—a positive potential observed approximately 300 ms after the stimulus presentation that reflects the formation of a link between stimulus evaluation and response selection (e.g., Petruo et al., 2016) —was more positive under stimulus-based conditions. However, the ERP mode was not included during the test phase. The interference effect of the reaction time between congruent and incongruent tasks that was discussed above was observed during the test phase, revealing the *effect-action retrieval* process involved in intention-based action. Therefore, the ERP mode must be investigated during the test phase in this study with the expectation of the interference effect of ERPs. According to previous studies, the contingent negative variation (CNV) component, which is thought to reflect action preparation that is identical to the RP component (Leuthold et al., 2004), tends to increase with the amount of advanced information provided by the precue, either because this information enables greater sufficient preparation for the following action (Leuthold et al., 2004) or because the subjects can access additional resources to complete the task (Falkenstein et al., 2003). In this study, we used a precuing task during the test phase to investigate the interference effect of CNV amplitude. We assumed that for intention-based actions, the CNV amplitude increases during congruent tasks because the congruent response rule facilitated action preparation more than the CNV amplitude during incongruent tasks because the incongruent response rule interfered with action preparation. This difference in CNV amplitude between congruent and incongruent tasks might be observed with intention-based action but not with stimulus-based action.

Based on these points, we can see that executive function and associative memory are involved in the cognitive processing of action. A growing number of studies have examined the training benefits of executive function (Karbach and Verhaeghen, 2014 for a review; Kelly et al., 2014 for a review) and associative memory (Derwinger et al., 2003; Bottiroli and Cavallini, 2009; Gross et al., 2012 for a review). It has also been reported that cognitive training, combined with multiple components, produced a broader effect on multiple cognitive domains than single cognitive training (Cheng et al., 2012; Walton et al., 2014). Therefore, we aimed to use combined cognitive training—including both executive function and associative memory—in this study of older Chinese adults to improve their action functions, which were measured by the related cognitive processes under specific action modes. The action modes of intention-based and stimulus-based actions were measured electrophysiologically separately with the pre- and post-tests of neuropsychological tests, respectively. We assumed that combined cognitive training would benefit both actions in terms of cognitive processing, which is reflected by the training effects observed in the amplitudes of the related ERP indices. Enlarged amplitudes after training are expected in the RP and P3 components under intention-based and stimulus-based action modes, respectively, suggesting beneficial gain in either the action-preparation or stimulus-processing components. For the CNV component, the interference effect—the amplitude difference between congruent and incongruent tasks—is also expected in intention-based actions after training, suggesting an improvement in action-effect association. Additionally, the training effect correlation between the ERP amplitudes and cognitive performance will be analyzed for further investigation. We predicted that after training, the improved executive function may benefit both intention-based and stimulus-based actions because of the fundamental and extensive role of executive function that may occur during action preparation. However, improved associative memory was predicted to benefit intention-based action only based on its specific role in action-effect association. In summary, we hypothesized that (1) combined cognitive training would benefit both intention-based and stimulus-based actions in their ERP modes and (2) the increased performance in executive function may be responsible for the training benefits of both actions, whereas the increased performance in associative memory may benefit only intention-based action.

## Materials and Methods

### Participants

The participants were a subset of the subjects enrolled in a larger study that was conducted by the Institute of Psychology, Chinese Academy of Sciences called Cognitive Training for Healthy Older Adults: Combined Training versus Memory Training^1^. In the larger study, healthy older adults were enrolled from the local community through advertisements. Forty subjects were randomly divided into memory training group and combined cognitive training group, with 20 subjects included in each training group. Eighteen subjects were recruited later from the local community for the control group. All subjects were (1) right-handed, (2) scored ≥24 on the Mini Mental State Examination (MMSE, Folstein et al., 1975), (3) scored ≤16 on the Center for Epidemiologic Studies Depression Scale (CES-D, Roberts and Vernon, 1983), (4) had normal or corrected-to-normal vision and hearing, (5) had no history of severe psychiatric or neurological disease, and (6) did not use drugs that might have adversely affected cognition (i.e., benzodiazepines or antipsychotics). In this study, 34 subjects in the combined training group and control group volunteered to participate in the ERP study. Of those who completed the baseline assessments, six subjects dropped out of the study, and two subjects were rejected due to an ERP data collapse caused by electrode failure. In the present ERP study, there were 16 final subjects (*N* = 16) in the combined training group and ten final subjects (*N* = 10) in the control group.

All the subjects participated in both the acquisition and test phases. During the test phases, the subjects in each age group were divided into two subgroups that performed congruent or incongruent tasks. The order of the intention-/stimulus-based acquisition and the congruent/incongruent tasks was counterbalanced for each group. For the training group, eight subjects performed congruent tasks and eight subjects performed incongruent tasks. For the control group, six subjects performed congruent tasks and four subjects performed incongruent tasks.

As shown in **Table 1**, the subjects in both groups did not differ significantly (*p* > 0.05) in terms of age, gender, education, global cognition (MMSE) or depression (CES-D). All the subjects were given written informed consent and were financially reimbursed for their participation. The study was approved by the Ethics Committee of the institute of Psychology, Chinese Academy of Sciences.

**Table 1:** Demographic and cognitive characteristics.

	Training (*n* = 16) M (*SD*)	Control (*n* = 10) M (*SD*)	*P*-value
Age (years)	69.6 (4.6)	69.0 (3.4)	0.74
Education (years)	12.4 (3.5)	13.1 (3.0)	0.62
Female/Male	9/7	6/4	0.85
MMSE	27.7 (1.5)	28.3 (1.5)	0.32
CES-D	4.6 (4.3)	3.7 (4.5)	0.60

### Procedures and Tasks

Both groups of subjects received cognitive assessments and ERP recordings of two action modes before and after training. The cognitive assessments and ERP recordings were conducted in two separate sessions that lasted approximately 100 and 150 min, respectively. The pre-training assessments and recordings were conducted within 2 weeks before training, and the post-training assessments and recordings were conducted within 1 week after training. The combined cognitive training group received 16 training sessions over the course of approximately 6 weeks, including eight initial sessions of executive function training and eight sessions of memory strategy training thereafter, in which it was supposed that enhanced executive function may facilitate the effective utilization of memory strategy. Each session lasted approximately 60 min (50 min of training with a break of 10 min). The subjects in the training group were asked to come to the institute three times per week and received 16 h of training in total.

#### Combined Cognitive Training

The combined cognitive training and the outcome measures used in this study were part of another interventional study (Li et al., 2016) with fewer subjects who participated in the ERP study of the two action modes. The more detailed content of combined cognitive training and outcome measures were presented in supplements to this article because the main focus of this study was the ERP modes of the training effect on actions.

##### Executive function training

*Updating training*. The updating training was adapted from the keep-track task (Yntema and Mueser, 1962) that included word-updating and picture-updating tasks from identical training sessions. The subjects were instructed to continuously update the items (i.e., either words or pictures) of targeted categories (e.g., animals, clothes, vegetables, and fruits) and verbally report the last item of each category at the end of the trial. Task difficulty was self-adaptive based on each subject, and the difficulty was manipulated by varying the number of categories presented (2 or 3) and the number of items in each category (2, 3, or 4).

*Switching training*. The switching training was adapted from the task-switching paradigm (Kray and Lindenberger, 2000), including only mixed-task blocks (two-task and three-task switching). The subjects were instructed to input responses by pressing one of two keys based on the cues presented at the bottom of the screen indicating the switched response rule. Task difficulty was also self-adaptive to each subject by varying the number of subtasks (two and three) and the number of items in each subtask (8, 10, and 12).

##### Memory strategy training

*Method of loci*. The method of loci (Bower, 1970) was used to recall a list of names of common objects (such as fruits and animals) with a mental image. The subjects were required to imagine a familiar route with several landmarks and the words associated with these landmarks using a mental map. The subjects were then asked to retrieve the words by revisiting the landmarks in their brain. The size of the wordlist was initially set at 8 and gradually increased to 10, 12, 14, and 16 with the training.

*Face-name mnemonic*. The face-name mnemonic (Yesavage et al., 1983) was taught to subjects to build an association between prominent facial features and name. In the recall phase, the subjects were required to retrieve the related mental image of the learned association and then recall the name. The number of practiced face-name mnemonics began from 1 face and increased to 7 faces progressively.

##### Outcome measures

Both the trained and untrained tasks were measured in the pre- and post- assessments. The detailed description of outcome measures was included in the Supplements Materials.

The trained executive function tasks included word- and picture-updating tasks and the switching task. The untrained executive function included the Trail Making Test (B-A) (TMT (B-A)), the difference value between the reaction time of TMT-B and TMT-A (Reitan, 1955), the Stroop Test (Stroop, 1935) and the Backward Digit Span Task (from the Wechsler Adult Intelligence Scale-Revised in China; Gong, 1992).

Trained memory tasks included the wordlist task and face-name task. The untrained memory task included the Associative Learning Test (ALT, from the Clinical Memory Scale, Xu and Wu, 1986) and the Logical Memory Test (LMT, from the Wechsler Memory Scale-Revised in China; Gong, 1989).

#### ERP Recordings

##### Apparatus and Stimuli

The subjects sat in front of a computer screen on a table that was 75 cm away from the subjects’ eyes. A keyboard with two response keys separated by a horizontal distance of 45 mm was placed on the table in front of the screen. A white cross (“+”) depicted on a black background at a visual angle of 0.8° × 0.8° served as the eye fixation site. Possible visual stimuli included the capitalized letters A, T, O, and X presented in white (height: 1.7°) against a black background at the center of the computer screen. During the test sessions, a centrally presented red asterisk (“^∗^”) with a visual angle of 0.8° × 0.8° was used as the imperative signal for a key press response. An auditory pacing signal consisting of sine tones (600 Hz; 100 ms in duration) was presented at the beginning of each block (see below) at a comfortable volume through mini-sound box sets placed on either side of the computer screen. E-prime (Version 2.0) software for Windows XP was used to present the visual stimuli and auditory signals to the subjects and to collect the behavioral data.

##### Tasks

**FIGURE 1:**
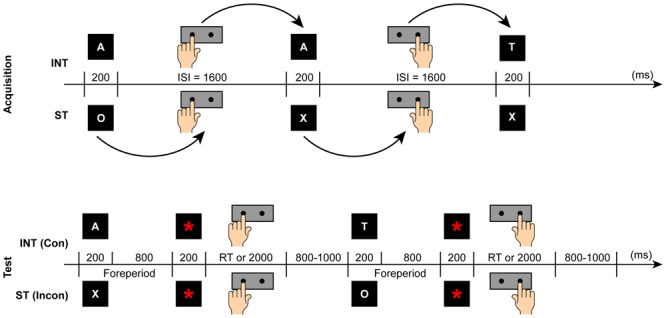
**Experimental procedure for the acquisition **(upper)** and test **(lower)** phases showing the trial sequence of intention-based (INT) and stimulus-based (ST) actions in congruent (Con) and incongruent (Incon) tasks.** ISI, Inter-Stimulus Interval.

Each subject completed four experimental sessions, including the acquisition and test phases, for both the intention-based and stimulus-based actions. As shown in **Figure 1**, all the subjects performed intention-based and stimulus-based acquisition during the acquisition phase. In other words, they pressed the left or right key either in a self-selected way or based on a specific rule. Following the acquisition phase, the subjects entered a test phase in which they were required to respond to the stimulus presented on the screen. During the test phase, half of the subjects performed a congruent task, and the remaining half performed an incongruent task. The order of the intention- and stimulus-based acquisition for the congruent and incongruent tasks was counterbalanced across each group.

##### Acquisition phase

A temporal bisection task adapted from Keller et al. (2006) was used during the acquisition phase. First, the subjects were presented with instructions and performed practice trials of the timed bisection *per se*, which was a pure timing task. This task required them to randomly press one of the two response keys to bisect a 1,600 ms time duration (i.e., the inter-stimulus interval, ISI) during which a fixation cross (“+”) was presented at the center of the screen, which was initiated by a centrally placed number eight (“8”) that appeared on the screen for 200 ms. Each practice block contained 20 trials, after which the subjects were apprised of their time performance to inform them of how frequently their responses fell within 350 ms of the exact bisection time point. The practice session ended when the subjects indicated that they were ready for the formal experiment and when their response accuracy rates were above the 80% criterion.

Electroencephalogram (EEG) electrodes were then fixed to the subject’s scalp. The subjects were then instructed concerning the performance of each type of action via both the visually presented instructions on the computer screen and a verbal explanation by an instructor. After a brief practice period, the subjects proceeded with the formal experiment. EEG recordings were collected during both intention-based and stimulus-based action sessions. As shown in **Figure 1**, in the intention-based acquisition session, the subjects were required to press the left or right key to bisect the 1,600 ms ISI. Each key press produced an “action effect” by determining the subsequent letter (i.e., A, T, O, or X) that appeared for 200 ms at the end of each ISI (e.g., the selection of the left key determined the appearance of A, and the selection of the right key determined the appearance of T). The subjects were instructed to attempt to implement a random sequence of letters, similar to tossing a coin, rather than to produce a sequence in a certain order. During the stimulus-based acquisition session, the subjects were required to press the left or right key at the bisection point of the ISI based on the rules associated with the appearance of a specific letter (A, T, O, or X; e.g., the appearance of O signaled a left key press, and the appearance of X signaled a right key press); these letters were presented in a randomized and counterbalanced sequence. To eliminate carry-over effects, different letters were presented for the same subject across the two actions.

Each block contained 40 trials, and the subjects were informed that they could take a break between blocks. For the first 10 trials of each block, an auditory pacing signal indicating the true ISI midpoint to assist the subjects with their timing performance. The subjects were required to synchronize their key presses with pacing signals and were told to try to maintain this rhythm when the auditory pacing signal ceased for the remaining 30 responses. As with the practice trials, in the experimental trials, the responses that were considered correct were within 350 ms (±350 ms) of the true bisection point. To ensure that a sufficient number of trials fit the ERP average under each condition, the blocks were repeated until 200 trials with correct responses (excluding auditory signaled trials) were performed.

##### Test phase

Upon completion of the acquisition phase, the participants proceeded to the test phase. Two sessions of the movement precueing task that were congruent or incongruent with the intention-based and stimulus-based acquisitions were administered. After the fixation cross (“+”) was presented for 200 ms, a letter serving as the precue (A, T, O, or X) appeared at the center of the screen for the next 200 ms to indicate whether the subsequent key pressed should be the left or right key. Additionally, the subjects were instructed not to respond directly to the precueing letter presented on the screen to ensure sufficient time for motor preparation before the motor response (Falkenstein et al., 2003; Leuthold et al., 2004). Although they already knew which key to press upon the appearance of the letter, they were instructed to withhold their responses until a red asterisk (“^∗^”) was presented (see **Figure 1**). This cue served as an imperative response signal and appeared at the center of the screen during the response period. Any responses occurring beyond this period were classified as incorrect. The response period ended with either a key press or ended after 2000 ms and was followed by a black screen for a random duration of between 800 and 1000 ms. Each test session consisted of 200 trials. The experiment ended after the two test sessions were completed.

During the test phase, the subjects were subdivided into two groups: one group performing congruent tasks and the other performing incongruent tasks. In the congruent task, the subjects were required to press a key based on the rules that they had learned during the acquisition phase. For instance, the subjects who had acquired left key press→A/right key press→T associations were then required to respond to A with a left key press and to T with a right key press. In the incongruent task, the subjects were required to perform the opposite key presses. For instance, the subjects who had acquired O→left key press/X→right key press associations were then required to respond to O with a right key press and to X with a left key press. The congruent and incongruent tasks described above are shown in **Figure 1**.

### Analysis

#### Analysis of the Cognitive Assessment Score

The composite scores were calculated separately for the executive function and memory measures to index the overall performance of these two cognitive functions. The raw scores for the five executive function tasks (word- and picture updating tasks, TMT (B-A), Stroop Test and backward digit span) and four memory tasks (wordlist task, face-name task, ALT and LMT) were standardized into *z* scores and then summed separately for the two cognitive functions and converted into a composite *t* score (*M* = 50, *SD* = 10).

Before the performance of one-way analysis of variance (ANOVA), the training and control groups’ baseline performances on the cognitive assessments were compared. Significant differences (*p* < 0.05) were found for the ALT, face-name task, memory composite *t*-score and backward digit span. Therefore, to control the baseline performance between the two groups, a covariance analysis (ANCOVA) was conducted on these four tasks. The posttest-minus-pretest change scores were compared between the two groups with the pretest scores on the above four tasks included as covariates. The ANCOVA was performed for each outcome measure and composite *t*-score. The switching task scores of the pretest from eight subjects in the control group were lost due to technical issues. Therefore, the switching task was not included in the analysis of this study.

#### ERP Recording and Data Analysis

The EEG data were recorded continuously from 64 cap-mounted Ag/AgCl electrodes (Neuroscan Inc.) arranged according to the International 10–20 System. The left mastoid served as an online reference, and the average of both mastoids was calculated oﬄine to enable algebraic re-referencing. The EEG data were amplified with a band-pass filter of.05–100 Hz and were digitized at 500 Hz. The vertical and horizontal electrooculograms (VEOG and HEOG, respectively) were recorded from two pairs of electrodes, one pair placed approximately 1 cm above and below the left eye and the other pair placed approximately 1 cm lateral to the outer canthi of both eyes. Inter-electrode impedances were maintained below 5 kΩ.

The EEG data were processed oﬄine, and the ocular artifacts were removed using a regression procedure (Semlitsch et al., 1986). The data were low-pass filtered using a cutoff frequency of 30 Hz. Epochs of RP were time-locked to the onset of a motor response and were segmented from a -900 ms pre-response to a 400 ms post-response, with -900 ms to -800 ms serving as the baseline period. Epochs of 700 ms (including the pre-stimulus baseline time of 100 ms) were extracted for P3, and 2,200 ms segments (including the pre-stimulus baseline time of 200 ms) were extracted for CNV; both were time-locked to the onset of the stimulus. Epochs exceeding ±100 μV were considered artifacts and excluded from further analysis.

For the acquisition phase, RP was measured as the mean amplitude at electrodes F3, Fz, F4, 384 C3, Cz, C4, P3, Pz, and P4 within a window of -400 to 0 ms because these signals reflect general motor preparation prior to action (Shibasaki and Hallett, 2006). The mean amplitude of P3 was measured within 400–500 ms over the centro-parietal sites (C3, Cz, C4, CP3, CPz, CP4, P3, Pz, and P4). Finally, for the test phase, the mean CNV amplitude was measured within 500–1,000 ms over the centro-parietal sites. The data were analyzed via a repeated-measures ANOVA considering time (pretest and posttest), action (intention-based and stimulus-based), anterior–posterior scalp location (anterior, medial, and posterior) and scalp laterality (left, middle, and right) as within-subject factors and group (training and control) as the between-subject factor. An analysis of the congruent and incongruent task subgroups was conducted exclusively for the CNV within the two groups while considering the task (congruent and incongruent) as a between-subject factor. Trials that were classified as incorrect were excluded from the ERP analysis. The Greenhouse-Geisser correction was used to adjust for sphericity violations. A *post hoc* analysis for significant main effects was performed using the Bonferroni method when necessary. Significant interactions were analyzed using simple effects models.

Correlation analyses between cognitive assessments and ERP mean amplitudes were also conducted when an ERP training effect was found. The change scores of the posttrain-minus-pretrain were calculated for cognitive assessments, except for the Stroop task and TMT (B-A). In these two tasks, the change scores of the pretrain-minus-posttrain were calculated because the lower scores indicated better performance. In calculating the ERP amplitude changes, the posttrain-minus-pretrain was used for positive ERP components, and the pretrain-minus-posttrain was used for negative ERP components, so that larger values indexed more enlarged amplitudes after training. Spearman’s rho was computed as two-tailed.

## Results

### Training Effects on Cognitive Assessments

**Table 2** shows the baseline performance, the means of change scores, significances and effect sizes (partial eta squared). The ANCOVA results showed significant training effects (*p* < 0.05) on the measure of the executive function composite score, word-updating and picture-updating tasks.

**Table 2:** Training effects on cognitive assessments of two groups.

	Training (*n* = 16)		Control (*n* = 10)		*P*^b^	Partial eta squared
	Baseline mean (SD)	Change mean (95% CI)	Baseline mean (SD)	Change mean (95% CI)		
**Executive function**
EF (composite t score)	48.6 (27.5)	10.37 (-4.60–25.35)	52.2 (23.0)	-16.20 (-35.14–2.74)	0.032	0.18
Stroop test^a^	11.6 (13.7)	-1.19 (-7.40–5.02)	16.6 (8.5)	-3.92 (-11.78–3.94)	0.579	0.01
TMT (B-A)^a^	32.0 (18.0)	-8.63 (-15.99--1.26)	26.9 (13.1)	0.09 (-9.22–9.42)	0.143	0.09
Digital span backward	4.3 (1.3)	0.38 (-0.19–0.95)	5.6 (1.4)	0.50 (-0.25–1.24)	0.807	0.00
Word updating	3.5 (2.3)	3.75 (2.71–4.79)	5.5 (2.7)	1.00 (-0.31–2.31)	0.002	0.90
Picture updating	6.0 (2.5)	2.94 (2.21–3.66)	6.0 (1.9)	1.60 (0.68–2.52)	0.027	0.19
**Memory**
Memory (composite *t* score)	41.1 (30.5)	4.48 (-6.84–15.80)	64.3 (15.9)	-7.16 (-21.80–7.47)	0.224	0.06
ALT	10.2 (2.3)	2.36 (0.65–4.06)	12.9 (2.5)	1.93 (-0.30–4.16)	0.770	0.00
Face-name	2.4 (1.9)	-0.11 (-0.84–0.62)	4.1 (2.0)	-0.18 (-1.12–0.76)	0.904	0.00
LMT	7.4 (2.6)	1.69 (0.89–2.49)	8.5 (2.1)	0.70 (-0.32–1.72)	0.128	0.09
Wordlist	8.9 (2.4)	1.50 (-0.48–3.48)	9.1 (1.7)	-0.20 (-2.70–2.30)	0.281	0.05

*SD, standard deviation; CI, confidence interval; ALT, Associative Learning Test; LMT, Logical Memory Test; EF, executive function; TMT, Trail Making Test. ^a^Lower scores indicate better performance. ^b^Analyses of covariance of posttraining-minus-baseline-change mean scores, controlled for baseline performance of backward digit span composite score of memory, ALT and face-name.*

### Training Effects on ERPs of Action Modes

The values of ERP indices under each condition was showed in **Table 3**.

**Table 3:** The means and 95% confidence interval (μV) of RP, P3, and CNV amplitudes in all conditions.

	Training group (*n* = 16)		Control group (*n* = 10)	
	Pretest mean (95%CI)	Posttest mean (95%CI)	Pretest mean (95%CI)	Posttest mean (95%CI)
**RP**				
INT	-0.57 (-1.42–0.27)	-0.44 (-1.52–0.64)	0.52 (0.72–1.76)	0.33 (-1.68–2.34)
ST	2.04 (0.96–3.11)	0.42 (-0.97–1.81)	2.65 (1.40–3.90)	3.33 (1.68–4.99)
**P3**				
INT	0.40 (0.09–0.71)	0.36 (-0.11–0.83)	-0.08 (-0.40–0.24)	-0.13 (-0.50–0.25)
ST	0.93 (0.54–1.32)	0.80 (0.24–1.36)	0.53 (-0.09–1.14)	0.20 (-0.61–1.02)
**CNV**				
INT-Con	-0.44 (-3.08–2.19)	-0.82 (-3.17–1.53)	-0.60 (-2.06–0.86)	-1.46 (-3.14–0.21)
INT-Incon	2.55 (-0.49–5.58)	0.42 (-1.39–2.22)	1.44 (-0.17–3.06)	-0.08 (-1.63–1.47)
ST-Con	-1.03 (-3.98–1.91)	0.53 (-1.92–2.99)	-0.92 (-2.35–0.51)	-0.74 (-1.91–0.43)
ST-Incon	1.75 (-0.86–3.86)	0.90 (-0.88–2.67)	0.08 (-1.07–1.24)	-0.66 (-1.96–0.64)

*CI, Confidence Interval; RP, readiness potential; CNV, contingent negative variation; INT, intention-based condition; ST, stimulus-based condition; -Con, congruent task; -Incon, incongruent task.*

#### Readiness Potential (-400–0 ms)

**FIGURE 2:**
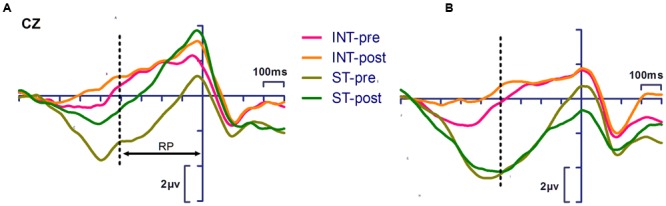
**Event-related potentials (ERP) waveforms showing significant time × action × group interaction (*p* < 0.05) of readiness potential (RP) amplitude of training group **(A**, left panel) and control group **(B**, right panel).** Simple effect analysis showed training effect of RP in stimulus-based action (*p* < 0.01). INT-pre, intention-based action in pretest; INT-post, intention-based action in posttest; ST-pre, stimulus-based action in pretest; ST-post, stimulus-based action in posttest. Response onset occurred at 0 ms. The dotted lines indicate measure time windows.

**Figure 2** presents the RP measurements of the pretest and posttest for both groups. There was a significant main effect of action [*F*_(1,24)_ = 40.325, *p* < 0.001, η_p_^2^ = 0.627], indicating that the RP amplitude for intention-based action was significantly more negative than that of the stimulus-based action. The interactions between time, action and group were also significant [*F*_(1,24)_ = 4.67, *p* = 0.041, η_p_^2^ = 0.163]. A simple effects analysis revealed that after training, the RP amplitude of stimulus-based action was significantly more negative (*p* = 0.005) than that of the baseline measure in the training group, but this measure was not significant in the control group (*p* = 0.387).

#### P3 (400–500 ms)

**FIGURE 3:**
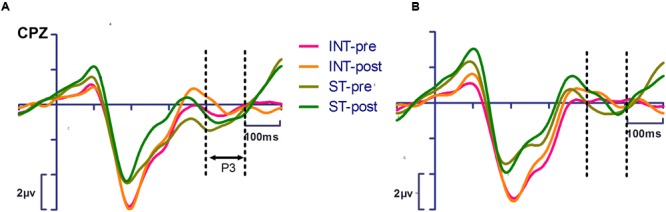
**Event-related potentials waveforms showing the P3 component of training group **(A**, left panel) and of control group **(B**, right panel).** No significant interaction was found for training effect. INT-pre, intention-based action in pretest; INT-post, intention-based action in posttest; ST-pre, stimulus-based action in pretest; ST-post, stimulus-based action in posttest. Visual stimulus onset occurred at 0 ms. The dotted lines indicate measure time windows.

The P3 measurements of the pretest and posttest are shown in **Figure 3** for both the training and control groups. There was a significant main effect of action [*F*_(1,24)_ = 11.487, *p* = 0.002, η_p_^2^ = 0.324], indicating that the P3 amplitude for stimulus-based action was significantly more positive than that for the intention-based action. No interaction was found to be significant between time, action and group.

#### Contingent Negative Variation (CNV) (500–1,000 ms)

**FIGURE 4:**
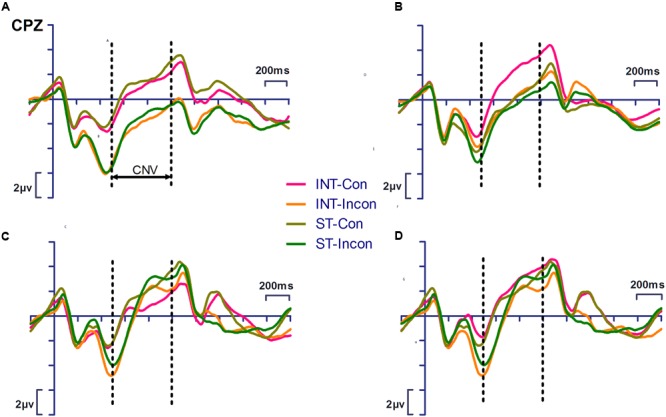
**Event-related potentials waveforms showing significant interaction of action × task (*p* < 0.05) of the contingent negative variation (CNV) elicited by congruent and incongruent response precueing tasks during the test phase in training group.** Simple effect analysis showed training effect of CNV amplitude in congruent task in intention-based action (*p* < 0.001). INT-Con, intention-based action and congruent task; INT-Incon, intention-based action and incongruent task; ST-Con, stimulus-based action and congruent task; ST-Incon, stimulus-based action and incongruent task. **(A)** the pretest of training group; **(B)** the posttest of training group; **(C)** the pretest of control group, **(D)** the posttest of control group. Precueing stimulus onset was set at 0 ms. The dotted lines indicate measure time windows.

**Figure 4** shows the CNV measurements of the pretest and posttest for both groups, in which a significant interaction between time and action was found [*F*_(1,22)_ = 14.156, *p* = 0.001, η_p_^2^ = 0.392]. A simple effects analysis revealed that after training, the CNV amplitude of intention-based action was significantly more negative (*p* = 0.017) than that of the baseline measure for both groups. However, such an effect was not found for the stimulus-based action (*p* = 0.757). This training effect was further analyzed within each group. The significant interaction between time and action was found in only the training group with a larger effect size [*F*_(1,14)_ = 17.155, *p* = 0.001, η_p_^2^ = 0.551] and was not found in the control group.

According to our assumption, the interference effect of CNV, which was reflected by the amplitude difference between congruent and incongruent tasks, was expected for intention-based action rather than stimulus-based action after training. Therefore, the pattern of CNV amplitudes between the two tasks and action modes was investigated within each group during each measure time. For the pretest, no significance was found for the main effect or the interaction of the action and task for either group. For the posttest, there was a significant action main effect [*F*_(1,14)_ = 25.98, *p* < 0.001, η_p_^2^ = 0.65] in the training group, indicating that the CNV amplitude of intention-based action was more negative than that of stimulus-based action. A significant interaction was also found between action and task [*F*_(1,14)_ = 5.905, *p* = 0.029, η_p_^2^ = 0.297]. A simple effects analysis showed that, in contrast with our assumption, the CNV amplitude of intention-based action was significantly more negative than that of the stimulus-based action (*p* < 0.001) in the congruent task but not in the incongruent task (*p* = 0.08). No significance was found for the main effect or the interaction of action and task in the control group.

### Correlation Results

As revealed by the ANOVA analysis, two training effects reflected by the increased amplitudes of RP in the stimulus-based action and CNV of the intention-based action were found in the combined cognitive training group. Marginal significance was found for the correlation between the change scores of the picture-updating task and the changed RP amplitude of stimulus-based action (*rs* = 0.482, *p* = 0.059), showing that improved performance of executive function after training was positively related with the increased RP amplitude in stimulus-based action. Significant correlations were also found between the change scores of the composite executive function (*rs* = 0.738, *p* = 0.037), the Stroop task (*rs* = 0.719, *p* = 0.045) and the TMT (B-A) (*rs* = 0.857, *p* = 0.007) and the changed CNV amplitude of intention-based action in the congruent task, showing that improved performances of executive function after training were positively related with the increased CNV amplitude in congruent task in intention-based action during test phase. The change scores of the Stroop task (*rs* = -0.778, *p* = 0.023) were significantly correlated with the changed CNV amplitude of intention-based action in the incongruent tasks, showing that improved performances of executive function after training were negatively related with the decrease CNV amplitude in incongruent task in intention-based action.

## Discussion

The present study investigated the training effects of combined cognitive training on intention-based and stimulus-based actions using ERP indices, including RP, P3, and CNV components. The training effects showed significant improvements for executive function performance but not for memory performance, consisting with Li et al.’s (2016) study. In comparison with the baseline performance, enlarged amplitudes of RP components in stimulus-based action and in the CNV component in intention-based action were revealed after training. Correlation analysis indicated that the ERP training effects observed in this study were related to improved executive function performance.

Based on our assumptions, the combined cognitive function training used in this study benefits both executive function and memory. However, the results showed that only executive function but not memory benefited from the training. The possible reasons for this finding have been discussed in Li et al.’s (2016) article, addressing insufficient training hours for both cognitive functions. In Li et al.’s (2016) study, a comparable memory training group receiving 16 sessions of memory training in total demonstrated a significant training effect of memory, whereas the combined cognitive training group demonstrated a significant training effect for the executive function and marginal significance for memory. In this study, the same combined cognitive training group and control group were involved, with fewer participants completing the ERP tasks before and after training, revealing a significant training effect on executive function but not on memory.

The ERP results showed a significant main effect of action for both RP and P3 amplitudes during the acquisition phase. This result replicated the findings of previous studies with regard to the modal differences between the two types of actions and has been discussed in depth (Waszak et al., 2005; Keller et al., 2006). In other words, a higher RP amplitude was observed for intention-based action than for stimulus-based action, reflecting that the process of motor preparation was more prominent for the intention-based condition. A larger P3 amplitude was observed for stimulus-based actions than for intention-based actions, indicating that the process of stimulus evaluation and response selection was more prominent for the stimulus-based condition. This mode difference between two actions was consistently presented in this study for both groups before and after training, suggesting that the two action modes were acquired under experimental manipulation.

The training effects revealed by ERPs were investigated separately during the acquisition and test phases. Consistent with our assumption, during the acquisition phase, it was revealed that the RP amplitude of the stimulus-based action was enhanced after training in comparison to that of the baseline. Thus, the stimulus-based action was improved after cognitive training, which may be related to enhanced executive function. In previous studies, the relationship between executive function and motor preparation has been reported in older adults and the related brain activations in prefrontal cortex has been discussed (Sterr and Dean, 2008; Berchicci et al., 2012). Moreover, tasks indexing stimulus-based action such as reaction time task were mostly used in previous laboratory studies. Therefore, our result was consistent with previous studies. The training effect was not observed for the RP amplitude of intention-based action and the P3 amplitude of both actions. These results demonstrate that executive function training benefited motor preparation rather than stimulus processing. A previous ERP study (Berchicci et al., 2012) has revealed that the age-related decline in action was more pronounced for motor preparation than for stimulus processing. The over-recruitment of the prefrontal cortex (PFC) was observed during the motor preparation for both simple and complex tasks in older adults compared to young adults. Therefore, it might be that the executive function training in this study enhanced the activities of the PFC in older adults, which improved the motor preparation of the stimulus-based action. The relationship between executive function and motor preparation has been demonstrated in previous studies (Sterr and Dean, 2008; Berchicci et al., 2012; Woods et al., 2015). However, it is notable that the motor preparation of intention-based action in this study did not benefit from the cognitive training. One possible explanation might be that the intention-based action was more complex and that older adults favor simpler stimulus-based action to show the age-related decline. The other explanation, according to our assumption, was that associative memory was responsible for the action-effect binding of intention-based action during the acquisition phase. The absence of the memory training effect in this study may account for the lack of improvement of intention-based actions.

During the test phase, based on the results and grand average of the CNV component, the training effect was mainly demonstrated as an enlarged amplitude of intention-based action in congruent tasks, which correlated with the changed scores of executive function after training and suggested that improved executive function was responsible for improved intention-based action after the combined cognitive training. It has been demonstrated in previous studies that the executive function was responsible for motor preparation (Sterr and Dean, 2008; Berchicci et al., 2012) and gained the greatest cognitive benefits from physical training in older adults (Colcombe and Kramer, 2003 for a review). Therefore, executive function may play a fundamental and extensive role in motor preparation. In the present study, we observed that improved executive functions after cognitive training were responsible for improvements in both intention-based action, which was indexed by an enlarged CNV amplitude, and stimulus-based action, which was indexed by an enlarged RP amplitude. Thus, consistent with our assumption, the improved executive functions benefits both intention-based and stimulus-based actions and provides evidence for the feasibility that action might gain benefits from cognitive training.

Nevertheless, the training effect of CNV amplitude was inconsistent with our assumption. The interference between congruent and incongruent tasks reflected by the CNV amplitude was not observed as we assumed—in other words, for intention-based action, the CNV amplitude in congruent tasks enlarges with the facilitation effect whereas the CNV amplitude in incongruent tasks decreases with the interference effect. In another study focused on the age effects on two actions (Niu et al., submitted) in which a young age group was involved, the interference effect of CNV amplitude replicating the RT interference between congruent and incongruent tasks was found only in intention-based action for young adults but not for older adults. Therefore, the absence of interference effects of CNV indicated the deficit of intention-based action in older adults, which was probably related to memory deficit in older adults.

According to the dual-process theory of memory, memory judgment is controlled either by the retrieval of specific details (i.e., recollection) or by the general strength (i.e., familiarity) of information (Rhodes et al., 2008). As suggested by the recall-to-reject theory (Rotello and Heit, 2000; Gallo et al., 2004), for the ability to reject the rearranged pairs of old items from the learned intact pairs, recollection was essentially required. For older adults, the recollection deficit is more sensitive to aging, and familiarity may become more favorable and reliable for associative memory retrieval (Naveh-Benjamin, 2000; Rhodes et al., 2008). As a result, older adults tend to endorse the rearranged pairs as intact pairs with high levels of false alarm observed, particularly when the items were repeatedly learned (Rhodes et al., 2008). In the present study, the effect-action association in the incongruent task involved a type of rearranged pair, in which the effects and action were learned repeatedly during the acquisition phase. Therefore, recollection should be necessary for the older participants to correctly recognize the rearranged association of effect and action that causes the interference effects. However, the recollection deficit of older adults may endorse the incongruent effect-action association, and the interference effect thus does not appear. In this study, although the improved executive function after training enlarged the CNV amplitude of the congruent task, unfortunately, memory performance did not benefit from the training, which might be responsible for the absence of the interference effect, which is reflected by the CNV amplitude of the incongruent task. Given more enduring memory training, a memory benefit may be gained—as shown in Li et al.’s (2016) study—and may hence improve recollection in older adults, which provides evidence for potential intervention targeting the improvement of intention-based action by means of cognitive training. In other words, although the executive function could benefit, the essential improvement of intention-based action may require the sufficient and enduring training of associative memory.

Previous studies have suggested that extensive executive function training could benefit the performance of episodic memory despite the small transfer effect (Buschkuehl et al., 2008; Dahlin et al., 2008). The brain activities involved in associative memory includes both the PFC and the medial temporal lobe (Naveh-Benjamin, 2000; see Yonelinas, 2002 for a review; see Blumenfeld and Ranganath, 2007 for a review). It was also demonstrated that memory strategy utilization required the involvement of executive function (Nyberg et al., 2003; Jones et al., 2006). The interaction between executive function and memory may indicate that although associative memory was responsible for the bidirectional association of action-effect in a specific manner, executive function may also play an important role in intention-based action. As shown in the results of this study, improved executive functions were related to either enlarged CNV amplitudes in congruent tasks or decreased CNV amplitudes in incongruent tasks, which appeared to demonstrate that the interference effect of CNV amplitude was caused by improved executive function. However, the inhibitive function reflected by the Stroop task was designed to help the participants focus on the presently required task by excluding the interference of irrelevance, which may possibly reduce the interference effect between congruent and incongruent tasks. Therefore, we believe that the interference effect reflecting the bidirectional action-effect association is caused by other cognitive functions such as associative memory, based on previous research and our assumptions. Unfortunately, the training effect of memory were not covered in this study, which may account for the absent interference effect of CNV in intention-based action after training. Nevertheless, the improved executive function after training may mainly benefit intention-based action by means of the enlarged CNV amplitude in the congruent task.

Some limitations should be noted in this study. First, the training hours used for both cognitive functions were relatively short, which may lead to the absence of the memory training effect and hence hinder the demonstration of the correlation between associative memory and bidirectional action-effect association in intention-based action. Further studies targeting intention-based action may establish sufficient training time for memory practice to further investigate the correlation and brain mechanism of associative memory in intention-based action. Second, the sample size was small and the individual differences were relatively large because fewer participants completed both the pretest and posttest of ERP tasks and there was a high dropout rate in the control group, which led to a limitation in the statistical power of the test hypothesis. As the results, on one hand, some potential positive effects may not be significantly observed in this study. In Li et al.’s (2016) study, marginal significance was found for the training effect on composite memory scores after the combined cognitive training, whereas no significance was observed for such an effect in this study, which had fewer participants. On the other hand, the limit statistical power could not support the present discussion firmly. As a pilot study, what was more important, it provided a new perspective to investigate the relationship between age-related cognitive decline and action, and discussed the potential mechanism of associative memory underlying intention-based action. Further studies using large sample sizes are also required to reveal the relationship between associative memory and intention-based action.

In sum, the present study provided empirical evidence demonstrating that action could benefit from cognitive training. The potential relationships of executive function and associative memory were discussed in the context of specific action modes. As we assumed, combined cognitive training benefitted both intention-based and stimulus-based actions. The improved executive function may produce extensive benefits to both actions, whereas associative memory may be specifically beneficial to the bidirectional action-effect association of intention-based action. A comparison of the cognitive processes between intention-based and stimulus-based action was introduced in this study to investigate the age-related decline and the brain plasticity of motor cognition in older adults, which offers a new pathway to investigate age-related motor cognition (in addition to the present research concerning movement disorders such as motor slowness, gait balance disorders and motor discoordination).

## Author Contributions

Y-NN and JL conceptualized the design. Y-NN took part in the data collection and wrote the draft. X-YZ took part in the data collection and contributed to discussion. JL critically reviewed and edited the manuscript. J-NF took part in the data analysis.

## Conflict of Interest Statement

The authors declare that the research was conducted in the absence of any commercial or financial relationships that could be construed as a potential conflict of interest.

## Footnotes

1 http://www.chictr.org.cn/showproj.aspx?proj=13140
