# Supplementary material for: The ERP Effects of Combined Cognitive Training on Intention-Based and Stimulus-Based Actions in Older Chinese Adults
Source: Front Psychol. 2016 Oct 27;7:1670. doi: 10.3389/fpsyg.2016.01670 (PMC5081365; doi:10.3389/fpsyg.2016.01670)
Supplement: Supplementary file 1 [file Data_Sheet_1.DOCX]

Supplementary Material

The ERP Effects of Combined Cognitive Training on Intention-based and Stimulus-based Actions in Older Chinese Adults

**Ya-Nan Niu*, Xin-Yi Zhu, Juan Li*, and Jiang-Ning Fu**

**Correspondence:** Ya-Nan Niu and Juan Li

niuyn@psych.ac.cn or [lijuan@psych.ac.cn](mailto:lijuan@psych.ac.cn)

# Supplementary material -The combined cognitive training and the outcome measures

1. **Combined cognitive training:**
   1. **Executive function training**

**Updating training:** The trained updating task was the keep-track task (Yntema & Mueser, 1962). In this study, the keep-track task was divided into Word-updating and Picture-updating tasks. Both were involved equally in training sessions. In this task, trials of colored words and pictures from various semantic categories (e.g. animals, clothes, vegetables, and fruits) were presented serially at 2000 ms/per item with an interval of 500ms in random order. Participants were instructed to continuously update the items of targeted categories indicated by boxes at the bottom of the screen and verbally report the last item of each targeted category at the end of the trial. Each trial included two 178 distracting items beyond the targeted categories. Each task included 12 trials of words or pictures. Difficulty was manipulated by varying the number of categories presented (two or three) and the number of items in each category (2, 3, or 4). Participants started with the easiest task (two categories with 2 items per category). When easier tasks were finished, they were instructed to conduct the more difficult tasks along with training sessions.

**Switching training**: We used the task-switching paradigm (Kray & Lindenberger, 2000) modified to include mixed-task blocks only (two-task and three-task switching). In this task, participants were required to switch subtasks (two subtasks or three subtasks) on pseudorandom trials according to cues presented in two boxes at the bottom of the screen in each trial. Two-task switching training consisted of two types of training materials performed equally in the training phase — “food”and “poker.” For the “food” task, subtask A required participants to decide whether a picture showed a fruit or vegetable (fruit/vegetable), and subtask B whether a picture was on the left or the right of the screen (left/right). For the “poker” task, participants were required to decide whether a poker card depicted was red or black (red/black), or whether the number on the poker card was even or odd (even/odd). There were also two sorts of training materials performed equally in three-task switching training — “face” (male/female, elders/youngsters, and white/yellow) and “poker” (red/black, even/odd, and<5/>5). The same two response keys were used for all tasks. Difficulty was manipulated by varying the number of subtasks (two and three) and the number of items in each subtask (8, 10, and 12). The number of trials in each task varied according task difficulty, ranging from 16 trials (the easiest task) to 36 trials (the hardest task). Participants started with the easiest task (two subtasks with 8 items per subtask) and continued to more difficult ones along with training sessions.

- 1. **Memory strategy training**

**Method of loci***:* Participants practiced method of loci (Bower, 1970) in the Wordlist task. They were instructed to establish a well-known route with several landmarks and to associate words with those landmarks serially on a mental map. At recall, participants were instructed to mentally revisit the ordered landmarks to retrieve the words. In this study, the Wordlist task words were names of common objects (e.g. animals, fruits) and were read aloud to participants. The method of loci practice started from 8-word lists, and then gradually increased to 10-, 12-, 14-, and 16-word lists. During the final 9-16 sessions, participants practiced only 16-word lists.

**Face-name mnemonic***:* The face-name mnemonic (Yesavage, 1983) was used in the Face-name task. In this task, black-and-white photos of males or females with two-character Chinese names were visually presented to participants on a computer. They were instructed to identify a prominent facial feature, creating a visual association with the name, and then mentally connect the association with the prominent facial feature. At recall, participants needed to identify the prominent feature first, retrieve the related mental image, and then recall the name. For the first 8 memory strategy training sessions, participants progressively practiced the face-name mnemonic on 1-face, 2-face, and 3-face tasks. During sessions 9-16, participants kept practicing on 5- and 7-face tasks.

1. **Outcome measures**
   1. **Trained executive function tasks**

**Word/Picture updating task**: Word- and Picture-updating tasks were structurally similar to the trained updating tasks. There were six two-category trials (half 3 items/category, half 4 items/category) and six three-category trials (half 2 items/category, half 3 items/category) in this task with two distracting items in each trial. The dependent measure was the number of blocks where the last presented items of each category were correctly recalled (maximum score = 12).

**Switching task:** Switching task was structurally similar to the trained “poker” switching task. This task was three-task switching with 24 items per subtask. Switching costs, the reaction time difference between non-switch and switch items, were measured as dependent variables. Each trial began with fixation-cross presentation (2000ms) and did not advance until participant response. Participants were instructed to respond as quickly and accurately as possible.

- 1. **Non-trained executive function tasks**

**Trail Making Test (B-A)**: The Trail Making Test (TMT, Reitan, 1955) included two parts. Part A is a neuropsychological test of processing speed and part B is a test of switching. Performance was indexed by the difference value between the reaction time on TMT-B and time on TMT-A (TMT B-A). Larger values in TMT (B-A) indicated poorer performance.

**Stroop Test** (Stroop, 1935): Participants were asked to name the color of dots/words/color words in three cards. Performance was indexed by the time difference between the card of color words and the card of dots. Larger values indicated poorer performance.

**Backward Digit Span Task** (from the Wechsler Adults Intelligence Scale-Revised in China; Gong, 1992): Participants were asked to reversely repeat lists of digits after each auditory presentation. The digit span was the length of the longest list a participant could repeat.

- 1. **Trained memory tasks**

**Wordlist task:** An audio-taped list of 16 two-character words was presented to participants at a rate of 6 s per word. At the end of the presentation participants were required to verbally recall as many words on the list as possible with no order constraint. The words were from four semantic categories (fruits, vegetables, animals, clothes) with 4 words per category and were chosen from the Directed Memory Test (from the Clinical Memory Scale; Xu & Wu, 1986). The dependent measure was the number of correctly recalled words (maximum score = 16).

**Face-name task:** Participants were presented with 12 black-and-white photographs of faces balanced for sex and paired with two-character Chinese names at the bottom of the screen. Each photograph was shown on the computer for 30s. At recall, these faces were presented in a different order, and participants were asked to verbally report the names previously paired with those faces. The dependent measure was the number of correctly recalled names (maximum score = 12).

- 1. **Non-trained memory tasks**

**Associative Learning Test** (ALT, from the Clinical Memory Scale, Xu & Wu, 1986): Participants were required to study an audio-taped list of 12 pairs of nouns for 2s per pair. Half of the word pairs were not associated (e.g. teacher-railway) and half were semantically associated (e.g. sun-moon). The list was presented three times with different orders. After each study phase, participants were asked to recall the second noun when given the first noun as a cue after each trial. The cues were not presented in the same order as during the training phase. The number of correctly recalled nouns was the dependent measure (0.5 point per pair for semantically-associated pairs, 1 point per pair for non-associated pairs, maximum = 27).

**Logical Memory Test** (LMT, from the Wechsler Memory Scale-Revised in China; Gong, 1989): Participants were presented with two audio-taped stories and were asked to verbally recall a story immediately after its presentation. The dependent measure was the mean score of the number of correctly recalled episodes (50 in total) of two stories (maximum score = 25).
